# Trabectome success factors

**DOI:** 10.1097/MD.0000000000007061

**Published:** 2017-06-16

**Authors:** Constance O. Okeke, Eydie Miller-Ellis, Mario Rojas

**Affiliations:** a Eastern Virginia Medical School; b Virginia Eye Consultants, Norfolk, VA; c Scheie Eye Institute, University of Pennsylvania School of Medicine, Philadelphia, PA.

**Keywords:** glaucoma, microincision glaucoma surgery, patient selection, phacoemulsification cataract extraction, trabeculectomy ab interno, Trabectome surgery

## Abstract

Our objective is to investigate which factors and patient characteristics are associated with success in Trabectome surgery.

A total of 658 phakic cases with at least of 12 months follow-up were included in the analysis. Baseline demographics and medical data were collected. The main outcome measure was intraocular pressure (IOP), glaucoma medication (Rx), and secondary glaucoma surgery if any. Success was defined as IOP reduction of 20% or more from preoperative IOP and IOP < 21 mm Hg with no secondary surgery throughout the follow-up period. Risk factors for failure were determined by using univariate and multivariate cox regression.

At baseline, the average IOP was 23.6 ± 7.8 mm Hg and the average number of medications was 2.6 ± 1.3 for all cases. At 12 months, the average IOP was 16.0 ± 3.6 mm Hg (*P* < .01^∗^) and the average number of medications was 1.8 ± 1.3 (*P* < .01^∗^). Based on the result of multivariate cox regression model, we found that the Trabectome + Phaco (TP) and Trabectome alone (TA) group had a 94% and 79% survival rate at 12 months, respectively. TP cases had 78% lower risk of failure than TA (95% confidence interval [CI]: 54–89), diagnosis of pseudoexfoliation glaucoma had a 54% lower risk of failure than primary open angle glaucoma patients (95% CI: 1–78). Hispanics had an estimated hazard ratio that is 60% lower than Caucasians (95% CI: 18–80); 20% of TA cases and 3% of TP cases were required to undergo additional secondary surgery (*P* < .01).

Trabectome surgery, whether in combination with phacoemulsification cataract removal or stand alone, is associated with a significant reduction of IOP and glaucoma medication. Patients having a higher baseline IOP are expected to have a higher IOP reduction after Trabectome. Pseudoexfoliation glaucoma, combination with phacoemulsification cataract surgery and Hispanic race are factors associated with enhanced Trabectome survival.

## 1 Introduction

Glaucoma is the world's second-leading cause of irreversible blindness.^[1]^ Methods of lowering intraocular pressure (IOP) are the mainstay of treatment, as IOP is the only known modifiable risk factor that can slow the progression of glaucoma. Medications are typically started as first-line therapy followed by laser trabeculoplasty when drops alone fail to control eye pressures. The well-established gold standard for surgical treatment has been external filtering surgery, such as trabeculectomy and aqueous drainage implants, as they have great success in IOP lowering to low levels for long terms. However, these traditional surgical procedures can also have significant intraoperative and postoperative complications, which at times can lead to serious vision-threatening conditions. These pressing issues that can occur, despite a technically perfect surgery, have led to the developments of less traumatic surgical options.

A new movement to microincision glaucoma surgery (MIGS) has been on the rise during the last decade. Ab interno trabeculotomy with the Trabectome was first introduced commercially in 2006 for microsurgical treatment of adult and infantile glaucoma, and began the movement of MIGS.^[2]^ Trabectome surgery has been reported to be effective in lowering IOP, reducing the need for topical glaucoma medications, and has been shown to have an excellent safety profile.^[3,4]^ Our current traditional procedures attempt to bypass the trabecular meshwork (TM), which has been shown to be the anatomic location of greatest resistance to aqueous outflow.^[5]^ The Trabectome is a surgical option that directly treats the highest point of resistance by removing the juxtacanalicular TM and inner wall of Schlemm canal (SC), which allows for exposure of collector channels to direct aqueous flow out of SC.

When choosing any IOP lowering surgery, it is very important to pick the right procedure for the right patient. Assessment of the angle, type and stage of glaucoma, and IOP lowering goal are all important in choosing the most suitable procedure for each individual patient. Trabectome is an effective MIGS for early to moderate, as well as advanced glaucoma.^[6]^ Our objective in this study was to investigate which factors and characteristics in phakic glaucoma patients are associated with success in Trabectome surgical outcome.

## 2 Methods

This is a prospective nonrandomized cohort study and outcome analysis of phakic patients undergoing Trabectome surgery. Data for this study were collected with institutional review board approval, in accordance with the Declaration of Helsinki and the Health Insurance Portability and Accountability Act. Subjects were enrolled from the Trabectome Study Group database,^[6]^ a postmarket surveillance requirement for NeoMedix. Each surgeon who performed Trabectome surgery was trained and qualified to perform Trabectome surgery. Patients undergoing the Trabectome procedure were followed for at least 12 months in the study cohort. Patients who underwent Trabectome combined with phacoemulsification cataract surgery with intraocular lens implantation (Phaco) were previously diagnosed with visual impairment due to lens opacification and also had glaucoma comorbidity. Patients either received Trabectome alone (TA) or Trabectome combined with cataract extraction surgery by phacoemulsification (TP). In most combined surgeries, the Trabectome procedure was performed first, and cataract surgery second.

Recorded preoperative data included the following: patient demographic data, type of glaucoma, IOPs, number of glaucoma medications, visual field, cup-to-disc ratio, Schaffer angle grade, previous ocular surgery, and previous ocular surgical complications. Primary outcome measures included IOP, number of glaucoma medications, and need for secondary glaucoma surgeries in the follow-up period. Inclusion criteria were all phakic patients diagnosed with glaucoma with or without cataract with at least 12 months follow-up. A history of laser trabeculoplasty did not lead to exclusion. Patients were excluded if they had missing preoperative data.

### 2.1 Surgical technique

The surgical technique employed for patients undergoing the Trabectome procedure was consistent with those previously published.^[3]^ In brief, surgical steps for the Trabectome procedure are as follows: after topical antibiotic and anti-inflammatory drops are instilled in the eye, anesthesia is then obtained with topical lidocaine gel and preservative-free lidocaine 1% in the anterior chamber. A surgical goniolens is placed on the cornea after viscoelastic and the angle view is verified. The goniolens is removed, and a 1.7-mm temporal clear corneal incision is performed with a keratome. Continuous irrigation is activated and then the tip of the Trabectome hand-piece is introduced into the eye. The goniolens is replaced. The Trabectome tip is advanced across the anterior chamber and inserted through TM into SC just anterior to the scleral spur. The TM is then ablated at a power of 0.8 to 0.9 W for approximately 2 or more clock hours in a counter-clockwise direction, then 2 or more clock hours in the clockwise direction.

### 2.2 Statistical analysis strategy

IOP and glaucoma medication at each time point (preoperative [baseline], 1, 3, 6, and 12 months postoperatively) were measured and recorded. The Wilcoxon test was used to compare IOP and number of glaucoma medications to baseline. Baseline characteristics were compared by using Mann–Whitney *U* test and chi-squared test for continuous and categorical variables, respectively. In order to evaluate the effect of phacoemulsification on IOP reduction between TA and TP groups, a statistical matching method, coarsened exact matching (CEM)^[7,8]^ was used. In survival analysis, preoperative IOP was categorized into 3 groups: ≤20, 20 to 29, and ≥30 mm Hg (IOP group), while preoperative number of medications into 4 groups: 0 to 1, 2, 3, and ≥4 meds (Rx group). Univariate cox regression method was used to examine variables as single main-effect associations with survival for all variables in order to identify possible adjusters and confounders. Based on the results of the univariate cox regression analysis and literature review, multivariate cox regression was used and interactions terms were investigated. All the statistically significant variables in the univariate analysis and potential risk factors identified in previous literature were added into the multivariate cox regression model. Success was defined as IOP reduction of 20% or more from preoperative IOP and IOP < 21 mm Hg with no secondary surgery throughout the follow-up period. Analysis was performed using R statistical software (R project) and Excel (Microsoft program).

## 3 Results

A total of 658 phakic cases with 1 year follow-up were included in the analysis. The majorities of the patients were Caucasian, diagnosed with primary open angle glaucoma (POAG), and had a Shaffer angle grade of 3 to 4. Please see Table 1 for the detailed demographic data and descriptive statistics on all patients. The demographics of the patients were analyzed by univariate cox regression to assess if the covariates have association with success and failure rate. From univariate analysis, age, race, diagnosis, IOP group, combination with cataract surgery, and Rx group were found to be associated with survival (Table 2). These factors, which had shown some degrees of association with survival, were screened using multivariate analysis.

**Table 1:**
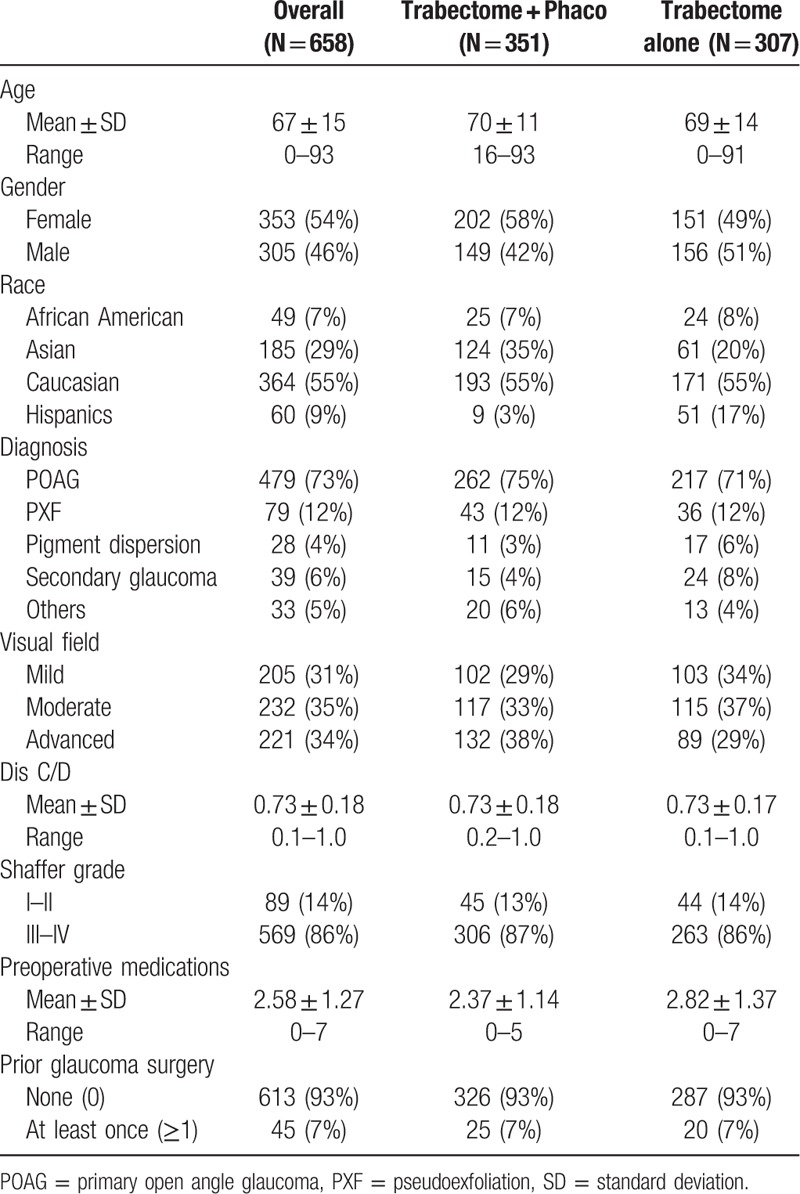
Demographics.

**Table 2:**
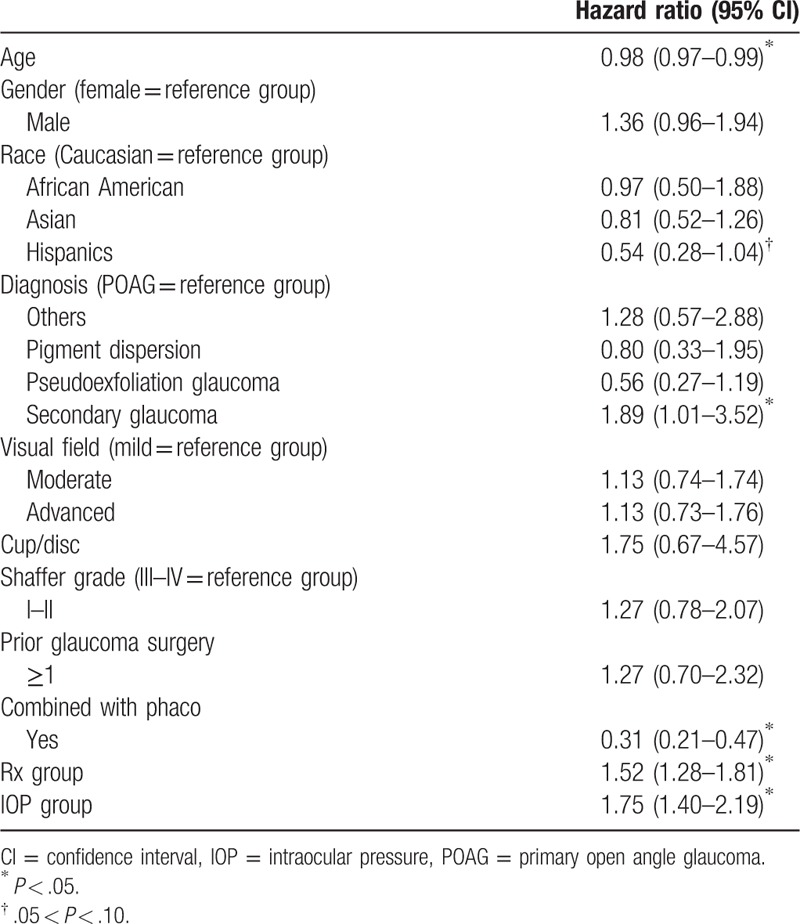
Summary of univariate survival analysis.

At baseline, the average IOP was 23.6 ± 7.8 mm Hg and the average number of medications was 2.6 ± 1.3 for all cases. At 12 months, the average IOP was 16.0 ± 3.6 mm Hg (*P* < .01^∗^) and the average number of medications was 1.8 ± 1.3 (*P* < .01^∗^). Based on the previous publication Mosaed^[9]^ patients were then subgrouped into TP (n = 351) and TA (n = 307) and then analyzed. The result is shown in Table 3. IOP for TP group was reduced from 21.0 ± 6.8 to 15.8 ± 3.4 mm Hg at 12 months (*P* < .01^∗^) and 26.5 ± 7.8 mm Hg to 16.3 ± 3.7 (*P* < .01^∗^) in TA group. Both groups showed statistically significant reduction in IOP from baseline at any follow-up time point. Statistically significant difference was found in mean change of IOP between TA and TP groups at 12 months. On average, IOP was reduced by about 5 mm Hg in TP group and 10 mm Hg in TA group at 12 months (*P* < .01^∗^). Both groups also showed statistically significant difference in number of medications compared with baseline. On average, both group reduced by 1 medication at 12 months and no statistically significant difference was found in medication reduction between groups.

**Table 3:**
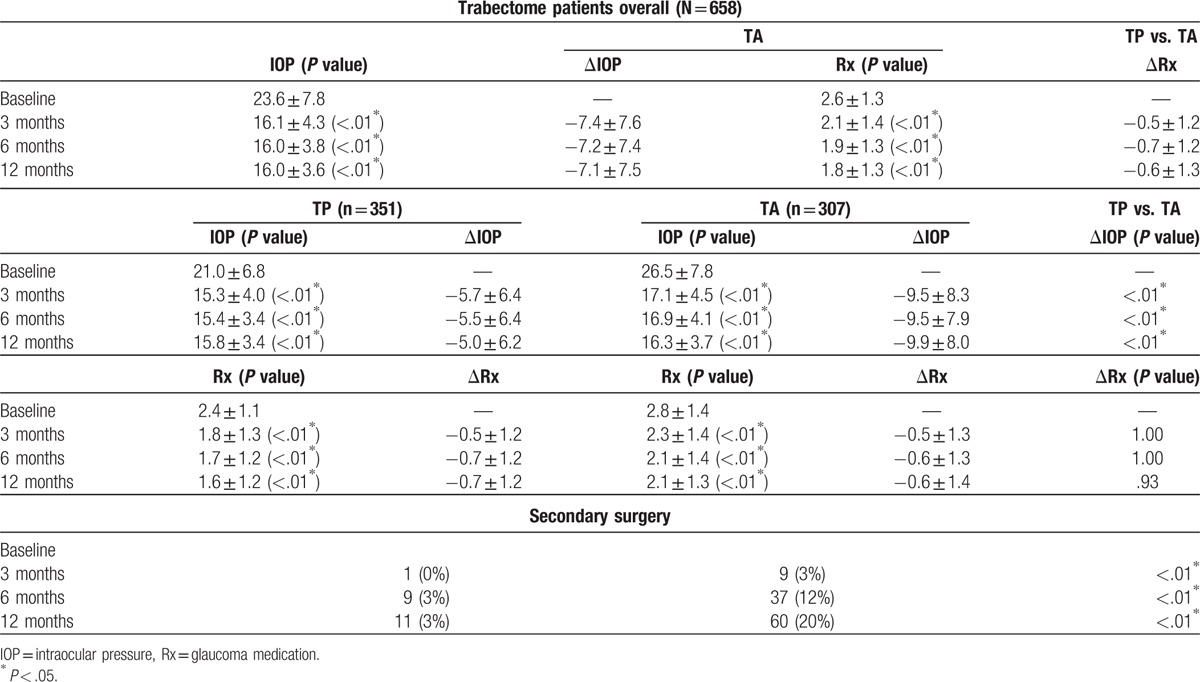
IOP, number of medications, and secondary surgery for phakic glaucoma patients undergoing Trabectome surgery: overall and subdivided to Trabectome alone (TA) and combined with Phaco (TP).

After matching and adjusting for age, baseline IOP, and number of medications, using CEM,^[7,8]^ Trabectome in combination with phacoemulsification had no diminished IOP reduction compared with TA and the difference in IOP was not found to be statistically significant. In other words, for patients with same age, baseline IOP, and number of medications, IOP reduction in TA patients was comparable to TP patients. Tables 3 and 4 show more TA cases (20%) required additional glaucoma surgery compared with TP (3%). No other statistically significant difference was found in complications between TP and TA (Table 4).

**Table 4:**
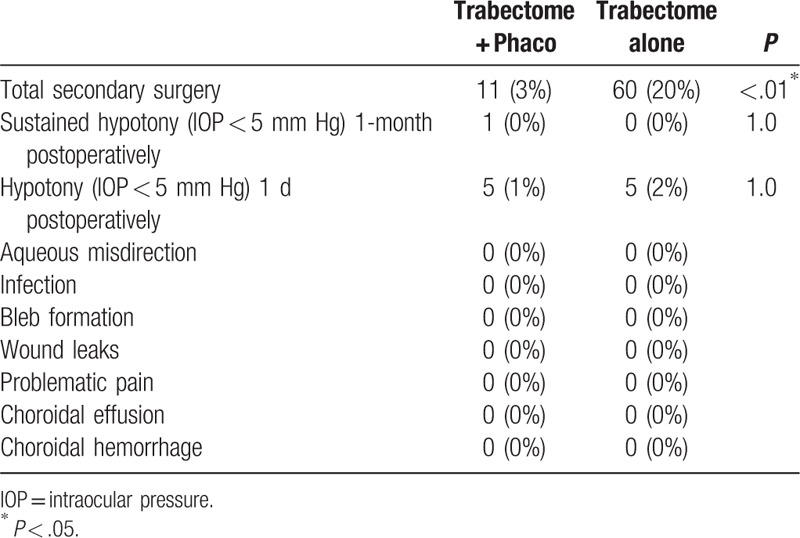
Postoperative observations at 12 months for phakic patients undergoing Trabectome surgery.

### 3.1 Results of cox regression model

**Figure 1:**
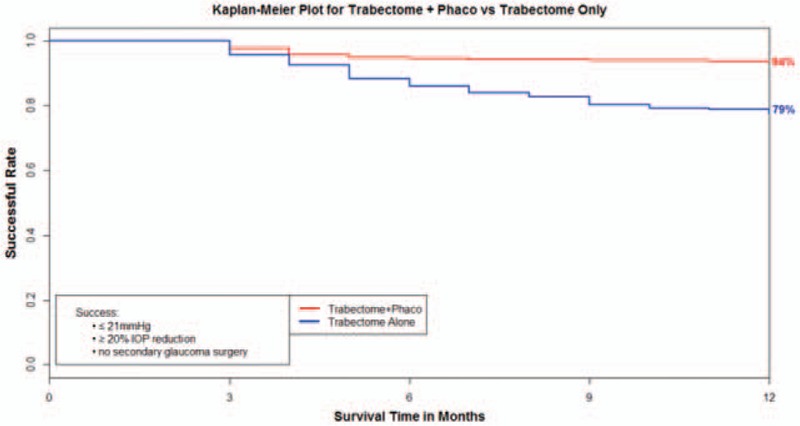
Based on the result of multivariate cox regression model, we found that the Trabectome + Phaco (TP) and Trabectome alone (TA) group had a 94% and 79% survival rate at 12 months, respectively.

**Figure 2:**
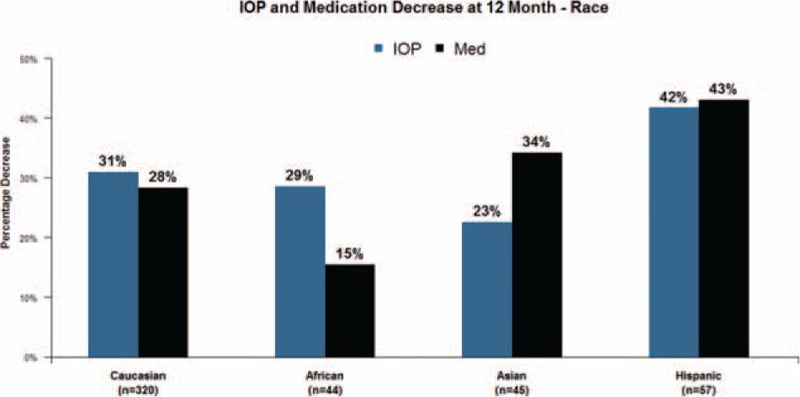
In total Trabectome cases, Hispanic race was found to be associated with a statistically significant reduction in intraocular pressure (IOP) and glaucoma medication compared with other races. Hispanics are expected to have 60% lower risk of failure than Caucasians (60%, 95% confidence interval: 18–80).

The multivariate model was stratified by Rx group, allowing different baseline hazard function for each category. The final model included age, combined with phacoemulsification, diagnosis, IOP group, race, and interaction between combined with phacoemulsification and IOP group (Table 5). Combined with phacoemulsification, pseudoexfoliation (PXF) glaucoma, and Hispanic race were found to be statistically significant, while interaction between phacoemulsification and IOP group was found to be marginally significant in association with Trabectome survival. After stratification by Rx groups and adjusted for diagnosis, race, and age, patients who had received TP are expected to have a lower risk for failure by 78% than those who had received TA in IOP Group 1 (78%, 95% confidence interval [CI]: 54–89). Based on the result of multivariate cox regression model, we found that each TP and TA group had a 94% and 79% survival rate at 12 months, respectively (Fig. 1). Adjusted for all other variables in the model, PXF patients are expected to have 54% lower risk of failure than POAG patients (54, 95% CI: 1–78) and Hispanic are expected to have 60% lower risk than Caucasians (60%, 95% CI: 18–80) (Fig. 2).

**Table 5:**
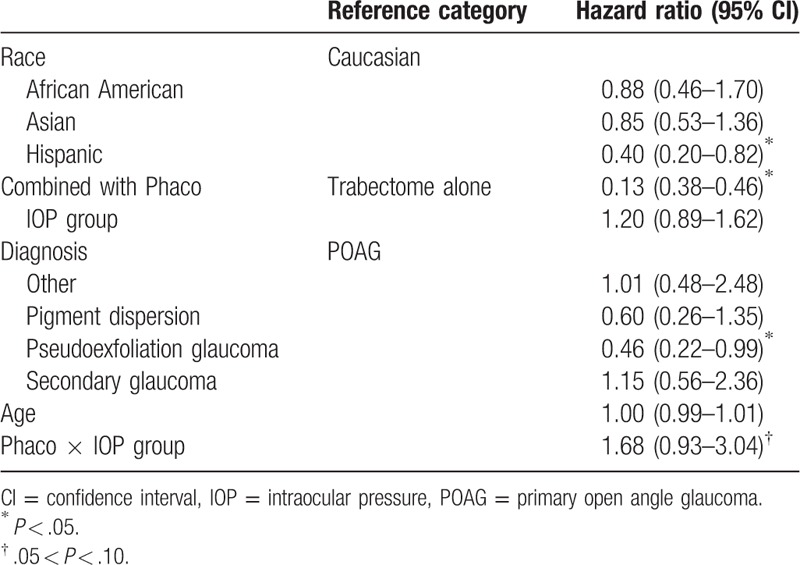
Final multivariate cox regression model.

## 4 Discussion

In this prospective, nonrandomized cohort study of Trabectome, we evaluated patient characteristics associated with success in Trabectome surgical procedures. Several factors in our multivariate regression analysis revealed an association with a higher rate of survival in Trabectome surgery: combination with phacoemulsification, diagnosis of pseudoexfoliation glaucoma, and Hispanic race. We also found that there was more need for additional glaucoma surgery in TA compared with TP, and that higher baseline IOP is associated with a larger IOP reduction postsurgery.

Our results show that diagnosed pseudoexfoliation glaucoma (PXF) patients have a 46% higher chance of successful outcomes compared with primary open angle (POAG) cases. Similar results have been reported in the literature. Ahuja et al^[10]^ reviewed 246 patients and defining success as postoperative IOP ≤ 18 mm Hg and ≥20% reduction. As they reviewed characteristics for success and failure, they also found that the PXF was associated with success as they reported a low hazard ratio for failure of 0.43 (0.27–0.67).^[10]^ Ting et al^[11]^ reported a 79% success rate at 12 months in PXF cases with an average of 40% reduction in IOP from baseline and 33% reduction in medications. Comparatively there was a 63% success rate in POAG cases with an average 28% reduction in IOP and 26% reduction of medications at 12 months.^[11]^

Jordan et al^[12]^ also reported a slight advantage with PXF patients having Trabectome compared with POAG. In 261 POAG eyes they found an overall IOP reduction of 25%, with a reduction in ocular hypotensive medication of 43%. For the 173 PXF eyes they found an overall IOP reduction of 30% with a simultaneous reduction in medication of 44%.^[12]^ Widder et al^[13]^ compared phacoemulsification and trabecular aspiration with and without the use of Trabectome (triple procedure) in PXF cases. Their results demonstrated average reduction of IOP to 38.4% in the triple procedure group and 26.8% in the control group without Trabectome (*P* = .004, and after Bonferroni correction, *P* = .008).^[13]^

Pseudoexfoliation is a disorder in which a fibrillar, proteinaceous substance is produced in high concentrations within ocular tissues and is the most common cause of secondary glaucoma worldwide, and frequent cause of unilateral glaucoma.^[14]^ In pseudoexfoliation glaucoma, pathogenesis involves clogging of the TM with the fibrillar material. The mechanism of action of the Trabectome involves removing a strip of TM and inner wall of SC. Therefore, the greater IOP lowering with Trabectome may reflect the mechanical removal of TM and exfoliation material wash out along with the irrigation and aspiration in and around the remaining TM.

The results in this study match established findings from other studies relating to larger IOP reduction with higher preintervention IOP. Vold compared the post 6-month outcomes of 1401 patients undergoing Trabectome procedures and classified them by baseline IOP levels. In eyes with IOP more than 30 mm Hg, reductions were 48% for IOP and 25% for glaucoma medications.^[15]^ In eyes with IOP between 23 and 29 mm Hg, reductions were 33% for IOP and 28% for glaucoma medications. In eyes with IOP between 18 and 22 mm Hg, reductions were 20% for IOP and 28% for glaucoma medications. Finally, in eyes with preoperative IOPs of ≤17 mm Hg, the mean reduction in IOP was 7%, with a 35% reduction in glaucoma medications. Minckler et al^[16]^ also reported similar findings in his case series of 1127 patients grouped as TA and TP. In their TA cases, the mean preoperative IOP was 25.7 ± 7.7 mm Hg and was reduced by 40% to 16.6 ± 4.0 mm Hg. In the TP cases, the baseline IOP was 20.0 ± 6.2 mm Hg and was reduced by 18% to 15.9 ± 3.3 mm Hg.^[16]^

We believe that these findings are due to several factors. In a preoperative setting, the indication for a patient who is having a Trabectome-only surgery is primarily motivated by need of IOP reduction to gain glaucoma control. In the case where the patient has a visually significant cataract as the driving factor for surgery, there is a mixed indication. The glaucoma is most often relatively stable with IOP controlled. The primary motivation is to improve vision and, if possible, reduce medications through the combined procedure and is less often the need for IOP reduction. This can explain why the TA cases tend to have higher overall IOP than TP cases. Also the IOP differences between TP and TA could also suggest an inherent disparity in the aggressiveness or severity of glaucoma in the TA and TP group, with the TA group possibly having more cases of aggressive or advanced glaucoma than the TP group. Dang et al^[17]^ evaluated Trabectome outcomes based on a glaucoma severity scale that included visual field, number of glaucoma medications, and preoperative IOP. They found that patients with higher pre-Trabectome IOPs who were in the higher glaucoma index group (more advanced glaucoma) had larger reductions of IOP.^[17]^

While Trabectome has the advantage of being a stand-alone procedure or one that can be combined with cataract surgery, our study supports the conclusion that either options can provide adequate pressure lowering. Both TA and TP had statistically significant reduction in IOP from baseline at 12 months with the TA group having a larger IOP reduction than the TP group. To further evaluate the IOP change disparity through the potential effect of phacoemulsification on IOP between TA versus TP, we used CEM to analyze this. After adjusting for age, baseline IOP, and baseline Rx, the IOP change disparity is lost in our matched analysis and combined with phacoemulsification was not found to be statistically significant. Thus, whether alone or in combination with Phaco, Trabectome can effectively lower IOP in phakic glaucoma patients. Also, on average there was a significant drop in medication usage that averaged about one in both groups. The significant differences in IOP and medication reduction in these groups have been similarly reported and efficacy of the procedure well established.^[3,4,13,18]^

Multivariate cox regression analysis found that Trabectome combined with Phaco is statistically significant risk factor associated with success. Kaplan–Meier survival analysis showed the TP group had a higher survival rate (94%) at 12 months than the TA group (79%). Thus, we conclude that TP group has a better survival rate than TA group. Other studies have also shown that the longevity of success with the Trabectome procedure is more often found with TP versus TA. In 290 eyes, Mosaed et al^[3]^ demonstrated that the success rate of TP cases was 87% with a 33% drop in medication usage, whereas in 538 eyes of TA cases at 1 year, the success rate was 65% with a 28% drop in medications. These findings were further supported in a large comparative cohort of 4659 Trabectome surgeries looking at primary outcomes.^[9]^ At 60 months, Mosaed et al found that the survival rate for TP was 85% compared to 58% with the TA group. In the cox regression model, we did not compare patients with and without Phaco, so we cannot say that phacoemulsification is causatively linked to a diminished IOP reduction. In our regression model, we compared patients with cataract who underwent TP and patients with cataract who underwent Trabectome only, and our findings do support that the longevity of successful IOP lowering seems to be better in the TP group versus the TA group.

Our results also showed that more TA cases (20%) underwent additional secondary surgery than TP cases (3%), despite the large decrease in IOP. These findings of TA cases having higher associations with long-term failure have been reported previously. In a study looking at extended follow-up, Minckler et al^[16]^ found that 14% of TA cases resulted in subsequent trabeculectomy or tube shunt surgery over 60 months of follow-up, while only 4% of the TP cases required it. Jordan et al^[12]^ reported that in a cox proportional hazards model correcting for age and lens-state, there was a significant superiority in terms of sparing further glaucoma surgery for the TP patients in comparison with TA patients in eyes being either phakic or pseudophakic at the time of operation. Jordan et al^[12]^ suggested that this result could be due to the additional IOP-lowering affect caused by the phaco alone. As mentioned above, these differences could be additionally explained by differences in the aggressiveness of the disease given the potential difference in main indication for surgery. Another explanation could be the development of peripheral anterior synechiae (PAS). Combining Trabectome surgery with phaco has the advantage of increasing the angle depth due to the smaller size of the intraocular lens which replaces the natural lens and subsequently widens the angle opening postoperatively. PAS has the theoretical potential to develop more often postoperatively in a phakic eye with TA, due to the proximity of the iris and corneal tissue that can scar due to inflammation, compared with the wider angle created with TP procedures.

The first paper introducing Trabectome as an MIGS procedure was done in a small patient population in Mexico in 2005.^[2]^ These 37 patients had great results, 27 of them self-defined Hispanics. Our results suggest that Hispanic patients had more successful outcomes when compared with Caucasians, African-Americans, and Asians. Support of this finding has also been recently reported in the literature. Dang et al^[17]^ showed that Hispanics had a larger IOP drop by 3.81 ± 1.08 than other ethnicities after Trabectome. Other previous studies may not have shown this advantage because there may not have been enough of a Hispanic population to demonstrate this.

Initial indications for the Trabectome called for use in open-angle glaucoma cases, and narrow-angle anatomy was a contraindication. One might assume that preoperative grading of the angle would be a strong indicator of success with the Trabectome. However, during the intraoperative surgical procedure, the angle anatomy is made more open and accessible with the increased pressure from the irrigation fluidics of the Trabectome device, thus allowing for successful completion of the procedure. In a combined procedure with cataract extraction, the angle remains open. In our univariate analysis, the data showed no statistically significant association with survival and angle width. These findings are supported by work done by Bussel et al,^[19]^ who specifically looked at the success of Trabectome with and without phacoemulsification and compared Schaffer grade of ≤2 and ≥3. After assessing 671 cases, they also did not find a statistically significant difference corresponding to preoperative Schaffer grading.^[19]^

Interestingly, our results, along with other investigations,^[12]^ have not shown a positive association of significant IOP reduction with pigment dispersion glaucoma. One can infer that removing the pigmented tissue from the TM with Trabectome would allow for improvement in outflow, and this can often be the case. But sometimes when the outcome is not as good as expected, there may be damage that extends beyond the TM in the more distal outflow system.

## 5 Study limitation

Our present study has identified certain patient characteristics that are associated with successful outcomes in Trabectome cases at 12 months. The limitation of this study is that it is not randomized. Randomizing the study will enable analyzing successful outcomes more precisely in patients with these characteristics after Trabectome surgery. Also, the 12-month follow-up length may be relatively short for following a chronic disease.

## 6 Conclusion

In conclusion, Trabectome surgery, whether in combination or alone, is associated with a significant reduction of IOP and medication. Patients having a higher baseline IOP are expected to have a larger IOP reduction. Factors associated with enhanced Trabectome survival include pseudoexfoliation glaucoma, combination with Phaco, and Hispanic race. These characteristics should help guide surgeons in the selection process to achieve optimum outcomes.

## Acknowledgment

The authors would like to acknowledge Ms. Jane Lin, Mr. Harry Gu, and Dr. Angele Nalbandian for their technical support.
